# New oral anti-coagulants versus vitamin K antagonists in high thromboembolic risk patients

**DOI:** 10.1371/journal.pone.0222762

**Published:** 2019-10-07

**Authors:** Annachiara Bellin, Patrizia Berto, Sakis Themistoclakis, Aastha Chandak, Pietro Giusti, Giacomo Cavalli, Sumeet Bakshi, Michele Tessarin, Paola Deambrosis, Alessandro Chinellato

**Affiliations:** 1 Università degli studi di Padova, Padova, Italia; 2 Analytica-Laser, a Certara company, Londra, United Kingdom; 3 LHU 3 Serenissima, Venezia, Italia; 4 LHU 2 Marca Trevigiana, Treviso, Italia; Inselspital Universitatsspital Bern, SWITZERLAND

## Abstract

**Background:**

Oral anticoagulant therapy (VKA) is nowadays the mainstay of treatment in primary and secondary stroke prevention in patients with atrial fibrillation. Given the limited risk-benefit ratio of vitamin K antagonists, pharmacological research has been directed towards the development of products that could overcome these limits, new oral anticoagulants were recently introduced: dabigatran, rivaroxaban, apixaban, and edoxaban.

**Aim:**

Scope of the present study was to examine patterns of use, effectiveness, safety and mean annual cost per patient of anticoagulant treatment for non-valvular AF in real clinical practice.

**Methods:**

A retrospective observational cohort study, by using administrative databases (drugs, hospitalizations, clinical visits, lab tests, population registry), was conducted in the Local Health Unit (LHU) of Treviso, Italy, from January 1, 2012 to December 31, 2016.

**Results:**

5597 subjects were selected, 2171 of which satisfied all inclusion criteria. In particular 1355 patients were treated with VKA, 577 patients were treated with NOAC, and 239 patients were treated initially with VKA and subsequently switched to NOAC (switch group). NOAC treatment showed to be superior to VKA and this superiority was statistically significant on both end-points: patients in the NOAC group reported less cardiovascular events (9,9%) and less bleeding episodes (5,5%) versus VKA patients (14,6% and 11,4%; p<,0001 and p = 0,0049, respectively).

The mean cost per patient per year was respectively € 1323,9 for patients treated with NOAC versus € 1003,3 for patients treated with VKA. Cost difference appears to be largely driven by drug cost (€ 767,9 for NOAC versus € 17,7 for VKA patients) and by specialist visits and laboratory tests (€ 318,4 for NOAC versus € 733,4 for VKA patients).

**Conclusion:**

In this retrospective real-world study treatment with NOAC showed to be associated with significant reductions of CV events and bleeding events compared to VKA use, albeit at a higher NHS’ direct cost per patient/year, mainly due to higher drug therapy cost.

## Introduction

Atrial fibrillation (AF) is the most common form of sustained arrhythmia in the clinical practice, and it is correlated with increased risk of cerebrovascular events and heart failure. A prevalence of about 1–2% of the population in western countries is reported, increasing with age and, at the same age, being higher in males than females [1]. Few observational registries have been developed to analyse the epidemiology of AF in Italy: the CUORE Project reports, in the general population, a prevalence of AF 1.0% and 0.7% for men and women, respectively. Furthermore, in people older than 65s (65–74), this parameter reached values of 2,5 and 2,4% for men a woman, respectively [2]. More recently, the FAI (Atrial Fibrillation in Italy) project showed a prevalence of FA of 7.3% for the whole over-65s GP-assisted population, with rates of 8.6 and 6.2%for males and females, respectively (this study was developed by the Neurofarba Department of the University of Florence, in 3 Operative Units located in Lombardy (Bergamo), Tuscany (Florence) and Calabria (Vibo Valentia), on all over-65s assisted by the participating GPs, for a total of about 6,000 subjects, 2,000 per Operative Unit) [3]. According to the Regional Epidemiology Department data, AF is estimated to affect 1.7% of the general population in Veneto (1.8 and 1.7% of males of females, respectively) with some variations between the Local Healthcare Units. Incidence is about 3/1000 person-years in the Region, increasing with age and male sex [4–5].

As population over 65 years will be increasing over the next few decades, an increase in the prevalence of AF is also to be expected. To date, the most frequent causes of AF are arterial hypertension (found in 50% to 65% of patients with AF), Diabetes Mellitus (15–20%), and hyperthyroidism (15%) [6].

Apart for diagnosis and initial therapy costs, the overall cost of AF is strongly influenced by chronic management of these patients: economic studies in this area showed that the greater weight of arrhythmia’s management costs is primarily due to the number and length of hospital admissions. A cost analysis of a UK registry of more than half million patients showed that hospitalisations and drug prescriptions accounted for 50% and 20% of the overall AF expenditure, respectively [7].

People with atrial fibrillation (AF) are more at risk of thromboembolic events; among these, the most fearsome (and potentially avoidable) event is cerebrovascular stroke: risk of stroke in patients with AF is 5 times greater than in patients on sinus rhythm [8].

The magnitude of risk for thrombo-systemic embolism (TSE) differs, depending on the aetiology of AF (whether valvular or non-valvular) as well as on coexistence of previous or current comorbidities. Oral anticoagulants and, to a lesser extent, antiplatelet agents (especially ASA in combination with clopidogrel), have been shown to be effective in significantly reducing thromboembolic strokes, even if associated with increased risk of bleeding [9].

Oral anticoagulant therapy (OAC) is nowadays the mainstay of treatment in primary and secondary stroke prevention in patients with AF. Given the limited risk-benefit ratio of vitamin K antagonists (VKAs), due especially to the higher bleeding risk, pharmacological research has been directed towards the development of products that could overcome this limitation, and the so-called new oral anticoagulants (NOAC) were recently introduced: dabigatran (Pradaxa**®**), rivaroxaban (Xarelto**®**) apixaban (Eliquis**®**), and edoxaban (Lixiana**®**) [10–13].

Scope of present study was to examine patterns of use, effectiveness, safety and average per patient cost of anticoagulant treatment for non-valvular AF (NVAF) in real clinical practice, based on retrospective claims databases from the Local Health Unit (LHU) of Treviso, Veneto, Italy.

## Materials and methods

### Study population

A retrospective observational cohort study was conducted at the Local Health Unit (LHU) of Treviso, Italy, from January 1, 2012 to December 31, 2016 using databases related to drug dispensing (pharmaceutical prescription), hospital or emergency admissions (discharged hospitalization or ER admission), population registry, laboratory test, and clinical visits (clinical blood test), covering an annual mean of 409,000 subjects (see Table A in S1 File) [14–15]. The eligible population included all subjects hospitalized from discharged hospitalization database between January 1, 2013 and December 31, 2015 with a diagnosis of atrial fibrillation (AF) (ICD-9 CM 427.3) (5,597 patients) and a prescription of anticoagulant therapy (2,903 patients), like warfarin or acenocoumarin and the new therapies (dabigatran, apixaban and rivaroxaban) from pharmaceutical prescription; excluding other anticoagulants such as heparin. All patients treated with NOAC were selected and a matched group of VKA patients was also extracted in a 2:1 ratio to NOAC, with CHA_2_DS_2_-VASC pairing criterion.

**Fig 1:**
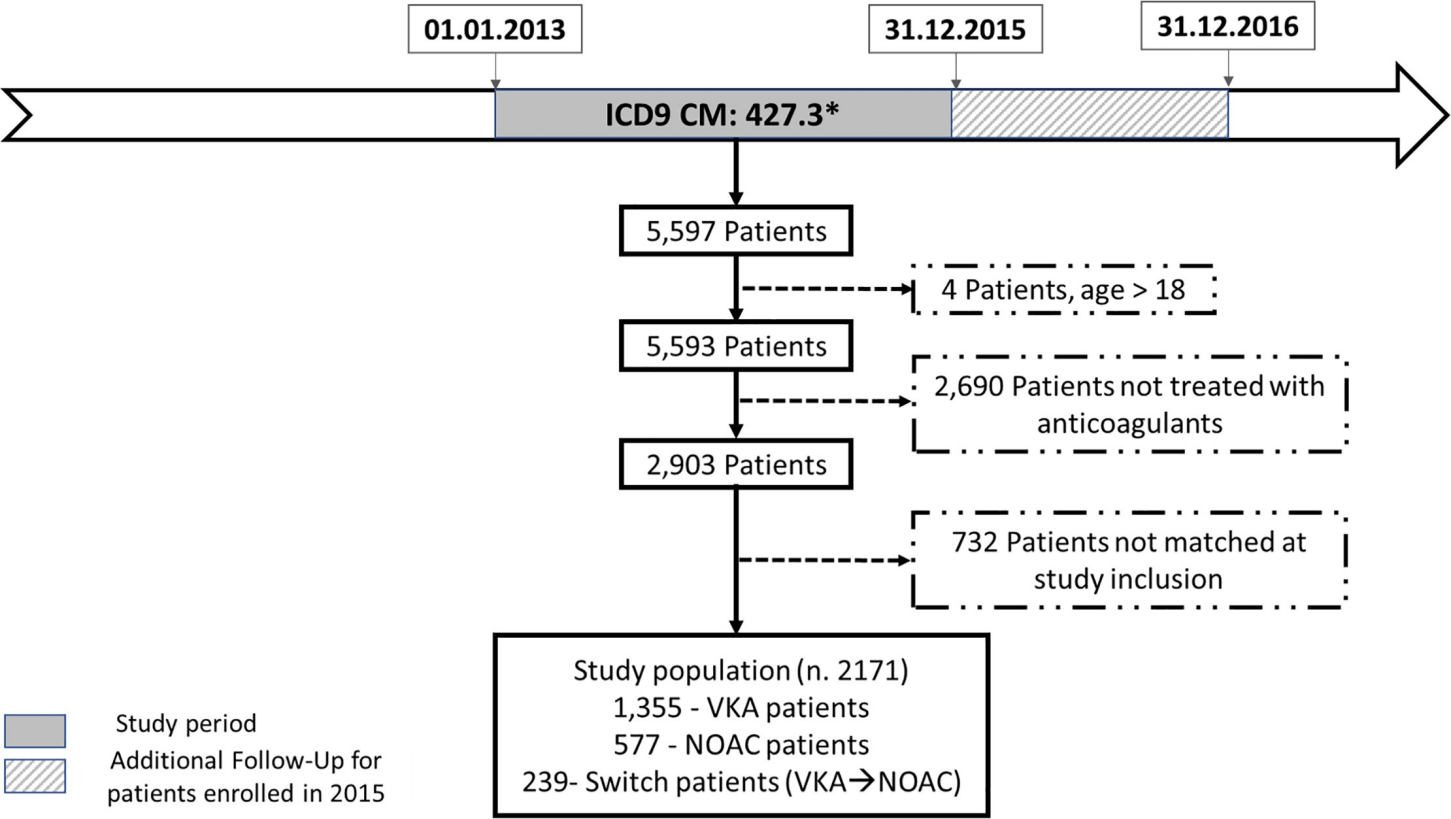
Flowchart showing selection of study population.

Study follow-up was extended to the end of year 2016, in order to account for patients enrolled during 2015 (to ensure a minimum of twelve months follow-up, for all patients) (Fig 1). All information collected contains encrypted anonymized data on patient age, sex, prescriptions and any concomitant disease. In addition, international normalized ratio (INR) parameters and clinical history of each patient were also extracted from the same databases (clinical blood test) (Table A in S1 File).

Individual stroke risk for each patient was defined according to CHA_2_DS_2_-VASC scores [16] and so CHA2DS2-VASC scores were calculated using administrative database on: registry population data (sex and age), discharged hospitalization, ER admission (previous stroke, heart failure, vascular disease), pharmaceutical prescriptions (antihypertensives, antidiabetics). Additionally, details about data source can be found elsewhere [17]

Exclusion criteria comprised: patients younger than 18 years (4 patients), valvular AF (defined according to 2014 guidelines [18]), patients who died within 7 days from the diagnosis, patients who received anticoagulant therapy occasionally (= number of prescriptions covering less than 6 consecutive months), patients with CHA_2_DS_2_-VASC score <2 (since reimbursement criteria for NOAC, include only patient with CHA_2_DS_2_-VASC score > 2, patients with CHA_2_DS_2_-VASC score <2 in the VKA cohort where explicitly excluded), and any patients not matching pairing criterion with NOAC population (732 patients).

Prescriptions of antiplatelet agents were excluded according to previous finding from the same database population [17].

#### Study subgroups defined according to therapeutic categories

Three different populations with NVAF were defined (see Fig 1): VKA group—patients who received VKA treatment for at least 6 months and had INR tested at least three times (in Italy INR testing is prescribed by General Practitioners–GPs—, and reimbursed by the NHS), NOAC group—patients who received NOAC treatment for at least 6 months, Switch group–patients who received at least 12 months of combined NOAC or VKA treatment, with at least 6 months of VKA and at least 6 months of NOAC treatment.

All drugs were identified according to the international anatomical therapeutic chemical classification system (ATC code): Vitamin K antagonist users were identified through the prescription of warfarin or acenocoumarin (ATC B01AA); NOAC users were identified through the prescription of drugs classified with code ATC B01AE and B01AF. Edoxaban treatment was not considered because its period of observation was too short (in Italy, this drug was commercialized on August 4, 2016).

The flow chart of patients enrolled in the study is reported in Fig 1.

### Study outcomes

Clinical outcomes (i.e. effectiveness and safety) were assessed, for each patient, by the occurrence of at least one cardiovascular event (i.e. hospitalization and/or Emergency Room (ER) admission) identified with ICD9CM diagnosis code. In patients that did not have any cardiovascular event between 1.1.2013 and 31.12.2015, the drug used (VKA or NOAC) was effective and/or safe. The criteria to define effectiveness and safety, are shown in supplements section Table B and Table C in S1 File, respectively.

### Costs

Only direct costs were analysed and calculated as the average cost / year / patient, considering anticoagulant treatment (number of packs prescribed per patient, multiplied by unit cost per pack), monitoring costs and cost of managing clinical events (number of INR examinations, specialist visits, hospitalisations, rehabilitation, emergency visits and investigations and laboratory examinations).

All unitary costs were retrieved from the administrative databases of Treviso Healthcare Unit (i.e. Registry population, discharged hospitalization, ER admission, Pharmaceutical prescription, Clinical blood test), representing the true cost borne by the NHS in delivering the services.

### Statistical analysis

Baseline characteristics of the populations were reported by means or medians and standard deviations (SDs) for quantitative information and by frequencies and percentages for qualitative characteristics. Statistical differences were analysed by t-test or chi-square/Fisher’s exact test. To define possible associations between risk factors and occurrence of stroke, a preliminary univariate analysis was run. In order to estimate association measures adjusted for time to anticoagulation treatment a cox proportional hazards model was used, estimating hazard ratios and p-value for time to CV events and time to bleeding events. Statistical analysis was performed using SAS software version 9.4 with statistical significance being defined at p<0.05.

### Ethics statement

This is an observational, retrospective, non-interventional study. In agreement with current national law, the study protocol was notified to the Ethical Committee of Treviso and Belluno.

Written informed consent was not necessary as all data extracted from the database were fully anonymized prior to receiving the data set.

The funder “LASER ANALYTICA, a Certara company”, provided support in the form of salaries for authors [PB, SB, AC], but did not have any additional role in the study design, data collection and analysis, decision to publish, or preparation of the manuscript. The specific roles of these authors are articulated in the ‘author contributions’ section.

The Authors confirm that this commercial affiliation does not alter their adherence to all PLOS ONE policies on sharing data and materials.

## Results

**Fig 2:**
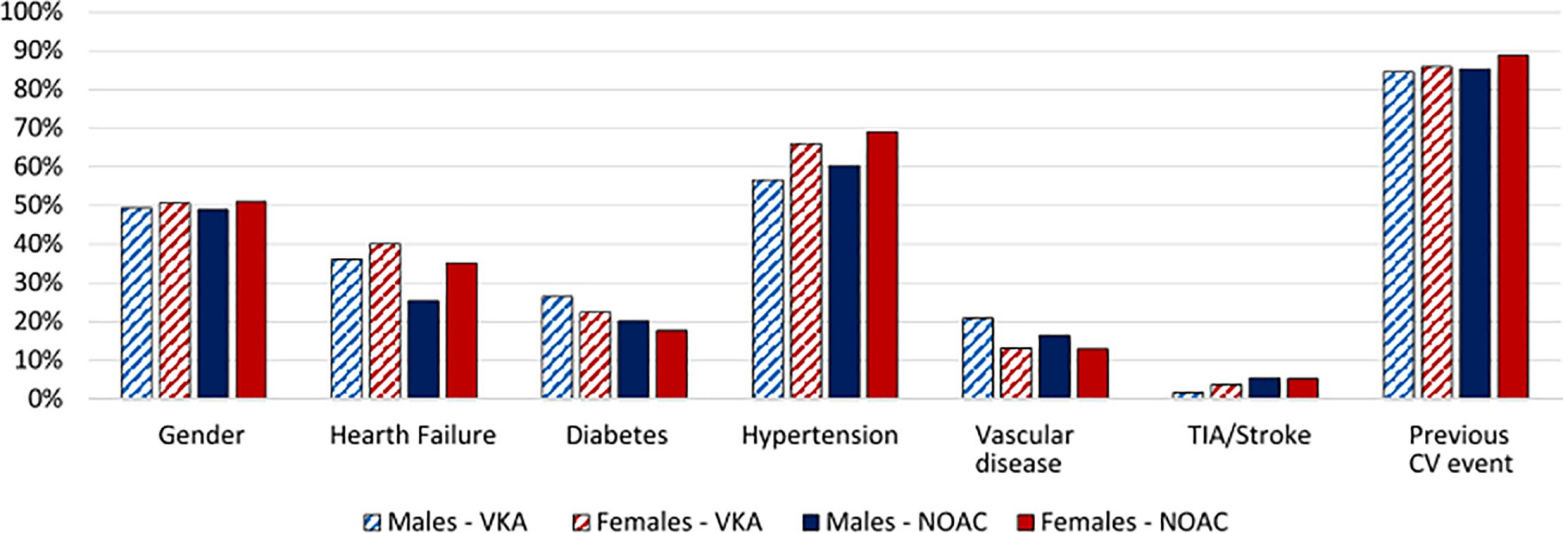
AF population characteristics.

In the two-year study selection timeframe (1.1.2013–31.12.2015), 5597 subjects (mean age 76± 12) from the population of Treviso LHU, were admitted to hospital or had an ER visit with a diagnosis of NVAF; population characteristics are illustrated in Figs 1 and 2.

Of the initial 5,597 subjects, 3,426 did not meet inclusion criteria, whereas 2,171 subjects satisfied all inclusion criteria. In particular, as shown in Table 1, 1,355 patients were treated with VKA (VKA group), 577 patients were treated with NOAC (NOAC group), and 239 patients were treated initially with VKA and subsequently switched to NOAC (switch group). No cases of patients who started treatment with NOAC and were subsequently switched to VKA, were found.

**Table 1:** AF treatment patient characteristics (VKA: VKA Group—NOAC: NOAC Group).

Group	VKA	NOAC	Switch patients on VKA+NOAC
**N of patients**	**1,355**	**577**	**239**
**Age, yrs**			
Mean (SD)	75.9 (8.6)	77.3 (9.2)	73.4 (8.1)
**Sex**			
Male, N	659	272	128
Male, %	48.6%	47.1%	53.6%
**CHA2DS2-VASc, score**			
Mean (SD)	3.1 (1.3)	3.1 (1.3)	2.9 (1.3)
**Previous TIA-Stroke**			
N	36	36	7
%	2.7%	6.2%	2.9%
P-value^¥^	-	0.0001*	0.8108

¥ Pair-wise P-value for comparison vs. VKA

*Statistically significant i.e. p<0,05

Average age, as well as sex distribution, were comparable across groups (Table 1); importantly, although the CHA_2_DS_2_-VASc score is similar across groups (as CHA_2_DS_2_-VASc ≥2 was one of patients’ selection criteria) the prevalence of TIA-stroke events before the pharmacological treatment was significantly different. In fact, it was 6.2 and 2.7% in the NOAC and VKA groups, respectively (p = 0.0001).

### Outcomes

Table 2 shows the results of the effectiveness and safety analysis: for both effectiveness and safety end-points, NOAC treatment showed to be superior to VKA and this superiority was statistically significant on both end-points: patients treated with NOAC (NOAC group) reported less cardiovascular events (9.9%) and less bleeding episodes (5.5%) versus VKA patients (14.6% and 11.4%; p<0.0001 and p = 0.0049, respectively).

**Table 2:** Patients with at least one CV or bleeding event, by treatment group (VKA: VKA Group—NOAC: NOAC Group).

Group	VKA	NOAC	Switch		
N of patients	1355	577	239		
			VKA+NOAC	Started with VKA	Proceed with NOAC
**Effectiveness end-point: CV events**					
N (%)	198 (14.6%)	57 (9.9%)	43 (18.0%)	32 (13.4%)	14 (5.9%)
P-value^¥^	-	0.0049*	0.1788	0.6197	0.0002*
Hazard ratio [95% CI] for comparison vs. VKA alone	1.0	0.9 [0.6–1.2]	1.1 [0.8–1.5]	1.2 [0.8–1.8]	0.5 [0.8–1.5]
P-value from Cox proportional hazards model for comparison vs. VKA alone	-	0.3425	0.5481	0.2773	0.0255
**Safety end-point: bleeding**					
N (%)	154 (11.4%)	32 (5.5%)	26 (10.9%)	17 (7.1%)	10 (4.2%)
P-value^¥^	-	<0.0001*	0.8265	0.0502	0.0008*
Hazard ratio [95% CI] for comparison vs. VKA alone	1.0	0.6 [0.4–0.9]	0.8 [0.5–1.4]	0.8 [0.5–1.4]	0.5 [0.3–1.0]
P-value from Cox proportional hazards model for comparison vs. VKA alone	-	0.0230	0.3612	0.5084	0.0419

^¥^ Pair-wise P-value for comparison vs. VKA

*Statistically significant i.e. p<0,05

The switch group (column 5 Table 2) (i.e, from VKA at least 6 months) failed to exhibit any significant difference compared to the VKA group either on the CV (p = 0.1788) and bleeding (p = 0.8265) events. However, after switching from VKA to NOAC treatments (see column 6 Table 2, ie NOAC patients) there was a significant improvement on both effectiveness and bleeding events as shown in Table 2: switch patients during VKA treatment period did not show improvement versus VKA alone (13.4%, p = 0.6197 for CV events and 7.1%, p = 0.0502 for bleeding events, respectively) whilst after switching to NOAC, for more than 6 months, significantly improved on both effectiveness and safety (5.9%, p = 0.0002 and 4.2%, 0.0008, for CV and bleeding events, respectively). Since follow-up time was not equal for all patients a cox-proportional hazards model has been used. So, in addition to the descriptive reporting through frequency and percentage, results from a cox proportional hazards model to estimate time to cardiovascular and bleeding events between the different treatment groups is included.

Patients treated with NOAC (NOAC group) had lower risk (HR = 0.6; 95% CI = 0.4–0.9; p-value = 0.0230) of experiencing bleeding episode as compared to VKA patients.

Switch patients during the VKA treatment period did not show any improvement versus VKA alone (p-value = 0.5481 for CV event and p-value = 0.3612 for bleeding episode) whilst after switching to NOAC had lower risk of CV event (HR = 0.5; 95% CI = 0.3–0.9; p-value = 0.0255) as well as lower risk of bleeding episode (HR = 0.5; 95% CI = 0.3–1.0; p-value = 0.0419).

#### Number needed to treat

Based on efficacy and safety outcomes the Number Needed to Treat (NNT) was calculated, representing the number of patients who need to be treated with NOAC versus VKA to avoid one cardiovascular event or one bleeding event. As shown in Table 3 the NNT for NOAC vs VKA is respectively 21.1 for CV events and 17.2 for bleeding events, meaning that every 21.1 patients treated with NOAC vs VKA, one CV event is saved or that every 17.2 patients treated with NOAC one bleeding event is saved.

**Table 3:** Number Needed to Treat NOAC vs VKA (VKA: VKA Group—NOAC: NOAC Group).

	Treatment Groups		NNT
	VKA alone	NOAC alone	NOAC vs VKA
**Effectiveness end-point: CV events**			
%	14.6%	9.9%	21.1
**Safety end-point: bleeding**			
%	11.4%	5.5%	17.2

### Cost analysis

Cost analysis is reported in Table 4; as shown, the mean cost per patient per year was respectively € 1,323.9 for patients treated with NOAC versus € 1,003.3 for patients treated with VKA.

**Table 4:** Cost analysis: Mean cost per patient/year over study follow-up (VKA: VKA Group—NOAC: NOAC Group).

	VKA alone	NOAC alone	Switch		
			patients on VKA	patients on NOAC	patients on VKA+NOAC
**N of patients**	**1,355**	**577**	**239**	**239**	**239**
**Drug, €**					
Mean	17.7	767.9	19.1	762.5	386.9
SD	13.6	171.3	12.0	159.9	166.5
Median	16.8	755.0	18.0	748.2	374.8
Q1	10.8	683.9	10.5	674.5	260.5
Q3	23.1	846.8	26.7	845.7	507.1
**Specialist Visits and Lab Tests, €**					
Mean	733.4	318.4	253.6	408.6	233.8
SD	8,559.3	326.0	602.2	432.2	164.0
Median	207.5	207.0	118.9	267.4	194.2
Q1	133.8	110.9	57.4	151.7	125.0
Q3	321.7	414.4	264.2	501.6	297.8
**ER Visits, €**					
Mean	9.6	6.6	41.6	8.1	24.2
SD	38.4	33.5	295.0	60.3	153.4
Median	0.0	0.0	0.0	0.0	0.0
Q1	0.0	0.0	0.0	0.0	0.0
Q3	0.0	0.0	0.0	0.0	0.0
**Hospital admissions, €**					
Mean	**242.5**	**231.0**	174.5	113.0	156.6
SD	1,046.6	1,127.6	722.8	599.8	561.2
Median	0.0	0.0	0.0	0.0	0.0
Q1	0.0	0.0	0.0	0.0	0.0
Q3	0.0	0.0	0.0	0.0	0.0
**Total per patient/year, €**					
Mean	**1,003.3**	**1,323.9**	488.8	1,292.1	801.5
SD	8,610.1	1,179.3	1,003.3	739.3	641.9
Median	260.1	1,040.6	174.5	1,076.2	642.3
Q1	162.7	881.9	86.2	911.5	483.5
Q3	444.5	1,276.7	416.9	1,421.0	845.7

Cost difference appears to be largely driven by drug cost (€ 767.9 for NOAC versus € 17.7 for VKA patients) and by Specialist Visits and Lab Tests (€ 318.4 for NOAC versus € 733.4 for VKA patients) with very marginal influence on total cost per patient/year of both CV-related ER visits and hospital admissions (respectively, € 6.6 and € 231.0 for NOAC versus € 9.6 and € 242.5 for VKA patients).

## Discussion and main findings

To our knowledge, this is the first Italian retrospective “real world” observational study investigating the efficacy and the safety of NOAC therapy compared to VKA in NVAF patients affected by non-valvular atrial fibrillation.

The main findings of this study can be summarized as follows: 1) use of NOAC is associated with a significant reduction of CV events and bleeding events compared to VKA use, 2) patient treated with both drugs over the follow up period experienced less CV and bleeding events during NOAC than VKA treatment, with no increase in bleeding or CV event risk during the switch period 3) NHS’ direct cost per patient per year is higher with NOAC vs VKA treatment, mainly due to higher drug therapy cost.

### Clinical outcome

Several randomized clinical trials proved that NOAC therapy is either equal or superior to VKA therapy in terms of efficacy (i.e. reduction of CV events) as well as safety (i.e. bleeding events) [10–13]. However, it is known that often only highly selected patients are enrolled in clinical trials, with age and comorbidities that are not equally represented in the general population. Moreover, patients are usually closely monitored by the enrolling centres: as a consequence of the “extra-care” of these patients (additional clinical outpatients visits, blood test, better compliance), the risk profile can be potentially affected, resulting in fewer clinical events, not fully reflecting the real-world scenario. One of the aims of “real-world” observational studies is to fill the gap between randomized clinical trials and routine daily clinical practice; the vast majority of studies on the use of NOAC in routine clinical practice, indeed confirmed data from previous randomized clinical trials.

Data from the Danish National patient registry published by Banerjee et al showed that NOAC therapy in high thromboembolic risk patients is associated with both lower ischemic stroke and fewer bleeding events, regardless of baseline bleeding risk [19]. More recently, a Spanish observational study of over 1,300, optimally VKA anticoagulated patients, showed that NOAC therapy was associated with stroke risk reduction up to 0.53%/year and bleeding risk reduction up to 0.88%/year, with even higher reductions in patients at high thromboembolic risk [20].

A metanalysis of 28 observational studies comparing NOAC and VKA therapy in real world setting confirmed and strengthened previous RCT data of dabigatran, rivaroxaban and apixaban, showing that all three NOAC are associated with large reduction of intracranial hemorrhage, similar risk of ischemic stroke and ischemic stroke or systemic embolism [21].

Our data, regarding a “real world”, high thromboembolic risk population, are aligned with those previously reported, showing that NOAC therapy is associated with a significant reduction of both CV events and bleeding events.

### Switching from VKA to NOAC

Due to the favourable profile of NOAC therapy in terms of efficacy and patient compliance, switching from VKA to NOAC therapy is becoming common practice, especially in low TTR (target to treatment) patients. However, issues are emerging regarding the switching window, as potentially the switch to NOAC could increase the bleeding risk, if VKA washout is incomplete.

In our population the switch group did not present a higher bleeding rate due to the change in OAC therapy. Moreover, clinical outcomes during VKA and NOAC follow-up period for switch patients were comparable to the VKA alone and NOAC alone groups. Our data demonstrate the safety of the switching strategy and confirm, as expected, higher efficacy of NOAC therapy after switch from VKA. These data are also confirmed by the cox-proportional hazards model analysis, accounting for different lengths of time on anticoagulant therapy among patient subgroups.

Real world data are aligned with our results, as it was widely shown that there is no increase of bleeding risk during switch from VKA to NOAC therapy: in a large, retrospective, matched cohort study by Bouillon et al [22], there were no differences between bleeding risk in those remaining in VKA compared to those switching from VKA to NOAC (2% vs 1%, p:0.54). Safety of switching was also confirmed from published data from the Dresden NOAC registry, with a very low rate of both cardiovascular events or major bleeding at 30 days follow-up [23].

### Cost and cost-effectiveness

Not surprisingly, some conflicting data about the economics of NOAC therapy are found in the literature, reflecting different drug costs across countries, as well as different event-related health costs, due to country-specific health system organizations and economics.

In a post-hoc analysis of the NOAC clinical trial published by Deitelzweig et al [24] the annual medical cost of NOAC therapy was estimated to be lower by $ −179 for dabigatran, $ −89 for rivaroxaban, and $ −485 for apixaban respectively, versus warfarin. However, those data are derived from randomized clinical trials, thus introducing a potential bias due to the highly selected and well monitored populations.

A cost-effectiveness modelling study by Coyle et al, based on a Canadian population showed that the most cost-effective treatments were apixaban for patients with high thromboembolic risk and without previous stroke, and dabigatran 150 mg in patients with high thromboembolic risk and with previous stroke. In the study, the overall cost for warfarin was estimated to be $ 300 per year, versus NOAC therapy cost of $1,100/year [25].

A US cost-effectiveness RCT-based, analysis of NOAC therapy in high thromboembolic risk patients showed that apixaban 5mg, dabigatran 150 mg and rivaroxaban 20mg were all cost-effective alternatives to warfarin. In the study, warfarin had the lowest mean per patient lifetime cost ($ 77,813), followed by rivaroxaban 20 mg ($ 78,738), dabigatran 150 mg ($ 82,719), and apixaban 5 mg ($ 85,326); against these differences in lifetime costs (which should always be translated with care to other geographies and settings), apixaban 5 mg had the highest quality-adjusted life-years estimate at 8.47 (SD, 0.06), followed by dabigatran 150 mg (8.41 ± 0.07), rivaroxaban 20 mg (8.26 ± 0.06), and warfarin (7.97 ± 0.04) [26].

Similarly, an Italian cost-effectiveness modelling study showed that, from the Italian NHS perspective, NOACs are a cost-effective treatment for the prevention of stroke in patients of age ≥71 years with NVAF, yielding incremental cost-effectiveness ratios always below € 25000/quality-adjusted life-year (QALY), for all NOACs, irrespective of CHADS2 scores. Estimated lifetime cost per patient in this model ranged from € 12801, to €14138, for VKA and from €18224 (apixaban in CHADS2 = 2 patients) to €22,122 (dabigatran in CHADS2≤1 patients) for NOAC [27].

Recently another comprehensive study was based on a systematic literature review and Network Meta-Analysis (NMA) of all published RCTs in the prevention of stroke in patients with AF. The NMA was based on 23 randomised trials involving 94,656 patients across multiple geographies, showing that all NOACs reduced the risk of stroke or systemic embolism, compared to warfarin. The economic analysis was run in the perspective of the UK NHS showing much lower expected lifetime cost per patient, than the previous US RCT-based model, ranging from £ 23,064 for dabigatran 150 mg twice daily to £ 24,841 for rivaroxaban 20 mg once daily; quite surprisingly the lifetime cost per patient of warfarin fall in between this range (£ 24,481) mostly due to the high costs of monitoring and events management with VKA. In terms of overall cost-effectiveness of the alternatives the study found that of the currently available DOACs, apixaban ranked highest on the balance of efficacy, safety, and cost with a probability of being cost-effective close to 60% in the £20,000-£30,000 range of willingness to pay, which is the range generally considered by NICE [28].

Our data show that NOAC therapy is associated with a 32% increase in cost per person/year (1,000 Eur/year for warfarin and 1,300 Eur/year for NOAC), but more lengthy follow-up would be needed to provide insight into the long-term cost of NVAF and to allow a full pharmacoeconomic analysis.

## Conclusion

In high thromboembolic risk patients, in an Italian real-world setting scenario, the use of NOAC therapy is associated with a reduction of CV events and lower risk of bleeding in both naïve patients and patients who switched from VKA therapy. However, NOAC therapy still generates higher cost per patient, compared to warfarin.

### Study limitations

This study presents several limitations: the study presents a retrospective design, with population representative of a north-eastern city of Italy, and results might not be extrapolated to other Countries; due to the retrospective nature of the study, and in line with its real-world data collection objective, our population was not randomized with possible consequent differences between VKA and NOAC groups. The study cohort included only patients hospitalized for non-valvular AF during the reference study period. In force of these inclusion criteria, the cohort was representative only of patients affected by AF directly or indirectly leading to at least one episode of hospitalization. Thus, results from this study cannot be generalized to all outpatients suffering of milder forms of AF, managed by GPs or specialists without hospital admissions

Clinical outcomes of the study are based on ICD9CM codes analysis; as a consequence, there could be coding errors or inconsistency of codes and outcomes; possible overestimation of events cannot be ruled out with certainty. Adverse events recognition was based on hospital discharge database identification. Minor events that did not require hospitalization were possibly not detected.

Due to the relatively limited time-frame of the analysis and the characteristics of the data extraction process, full cost-effectiveness analysis was not possible, as well as a comparative analysis between the different NOACs.

## Supporting information

S1 File **Table A.** Definition database used **Table B.** ICD9CM-criteria used for the effectiveness end-point analysis **Table C**. ICD9CM-criteria used for the safety end-point analysis
(DOCX)
