# Supplementary material for: New oral anti-coagulants versus vitamin K antagonists in high thromboembolic risk patients
Source: PLoS One. 2019 Oct 7;14(10):e0222762. doi: 10.1371/journal.pone.0222762 (PMC6779249; doi:10.1371/journal.pone.0222762)
Supplement: S1 File — Table A. Definition database used Table B. ICD9CM-criteria used for the effectiveness end-point analysis Table C. ICD9CM-criteria used for the safety end-point analysis (DOCX) [file pone.0222762.s001.docx]

***Supplementation Table A.*** Definition database used

| DATABASE | DESCRIPTION | CONTAINED INFORMATION |
| --- | --- | --- |
| Registry population | Include all assisted patients registered in the population register of the Local Health Unit (LHU).  This register is validated by the national registry office.  The connection field with the other databases is the Fiscal Code. | Fiscal Code  Sex  Age |
| Discharged hospitalization | Includes all hospitalizations, describes the causes of hospitalization for each patient, the five main diagnoses, the dates of admission and discharge and the cost.  The database is generated by doctors and the hospital administration, and validated by the Veneto Region, also for administrative purposes.  The connection field with the other databases is the Fiscal Code. | Fiscal Code  Date of Admission  Code of Diagnosis (ICD9CM)  Cost |
| ER admission | Includes all hospitalizations, describes the causes of hospitalization for each patient, the main diagnoses, the dates of admission and discharge and the cost.  The database is generated by doctors and the hospital administration, and validated by the Veneto Region, also for administrative purposes.  The connection field with the other databases is the Fiscal Code. | Fiscal Code  Date of Admission  Code of Diagnosis (ICD9CM)  Cost |
| Pharmaceutical prescription | The database is generated by the pharmaceutical prescriptions of general practitioners and hospital specialists working in the LHU, and validated by the Veneto Region, also for administrative purposes.  The connection field with the other databases is the Fiscal Code. | Fiscal Code  Date of Prescription  ATC Code  Drug  Cost |
| Clinical blood test | the database includes all the blood tests of the assisted patients in the LHU, and validated by the Veneto Region, also for administrative purposes.  The connection field with the other databases is the Fiscal Code. | Fiscal Code  Test  Test Code  Result of Test  Cost |

***Supplemental Table B.*** ICD9CM-criteria used for the effectiveness end-point analysis.

| CODE | DESCRIPTION |
| --- | --- |
| 336.1 | Unspecific Hematoma |
| 410* | Acute Myocardial Infarction |
| 411.1 | Other Acute and Subacute Forms Of Ischemic Heart Disease |
| 411.81 | Acute Coronary Occlusion Without Myocardial Infarction |
| 430* | Subarachnoid Haemorrhage |
| 431* | Intracerebral Haemorrhage |
| 432* | Other and Unspecified Intracranial Haemorrhage |
| 433.01-11-21-31-81-91 | Occlusion and Stenosis of Precerebral Arteries with Cerebral Infarction |
| 434.01-91 | Occlusion of Cerebral Arteries with Cerebral Infarction |
| 435.0-1-2-3-8-9 | Transient Cerebral Ischemia |
| 436* | Acute, But Ill-Defined, Cerebrovascular Disease |
| 444* | Arterial Embolism and Thrombosis |
| 445.0-01-02-81-89 | Atheroembolism |
| 453* | Embolus and Thrombosis |
| 798.98 | Death |
| 799.9 | Other Ill-Defined and Unknown Causes of Morbidity and Mortality |

***Supplemental Table C***. ICD9CM-criteria used for the safety end-point analysis

| CODE | Description |
| --- | --- |
| 360.43 | Hemophthalmias, Except Current Injury |
| 280.0 | Iron Deficiency Anaemias |
| 285.1 | Acute Post haemorrhagic Anaemia |
| 285.9 | Unspecific Anaemia |
| 362.43 | Haemorrhagic Detachment of Retinal Pigment Epithelium |
| 362.81 | Retinal Haemorrhage |
| 363.61 OR 363.62 OR 363.72 | Expulsive Choroidal Haemorrhage |
| 364.41 | Hyphema Of Iris and Ciliary Body |
| 372.72 | Conjunctival Haemorrhage |
| 374.81 | Haemorrhage of Eyelid |
| 376.32 | Orbital Haemorrhage |
| 377.42 | Haemorrhage in Optic Nerve Sheaths |
| 379.23 | Vitreous Haemorrhage |
| 423.0 | Hemopericardium |
| 432.2 | Other and Unspecified Intracranial Haemorrhage |
| 436.36 | Acute, But Ill-Defined, Cerebrovascular Disease |
| 444.3 | Endoscopic Control of Gastric or Duodenal Bleeding |
| 456.0 OR 456.2 | Oesophageal Varices with Bleeding |
| 459.0 | Haemorrhage, Unspecified |
| 530.7 | Gastroesophageal Laceration-Haemorrhage Syndrome |
| 530.82 | Oesophageal Haemorrhage |
| 531.0 OR 531.2 | Acute Gastric Ulcer with Haemorrhage |
| 531.4 | Chronic or Unspecified Gastric Ulcer with Haemorrhage |
| 532* | Acute Duodenal Ulcer with Haemorrhage |
| 532.40-41-60-61 | Chronic or Unspecified Duodenal Ulcer with Haemorrhage |
| 533* | Acute Peptic Ulcer of Unspecified Site with Haemorrhage |
| 534* | Acute Gastro jejunal Ulcer with Haemorrhage |
| 535.*1 | Acute Gastritis, With Haemorrhage |
| 537.83 | Angiodysplasia Of Stomach and Duodenum with Haemorrhage |
| 557.0 | Acute Vascular Insufficiency of Intestine |
| 562.02- 03-12-13- | Diverticulosis of Small Intestine with Haemorrhage |
| 568.81 | Hemoperitoneum (Nontraumatic) |
| 569.3 | Haemorrhage of Rectum and Anus |
| 569.85 | Angiodysplasia Of Intestine with Haemorrhage |
| 577.0 | Haemorrhagic Acute Pancreatitis |
| 578.1-78-9 | Haematemesis |
| 596.7 | Haemorrhage into Bladder Wall |
| 599.7 | Haematuria |
| 719* | Hemarthrosis |
| 782.7 | Spontaneous Ecchymoses |
| 784.7 | Epistaxis |
| 784.8 | Throat Bleed |
| 786.3 | Haemoptysis |
| 852.1* | Subarachnoid Haemorrhage Following Injury Without Mention of Open Intracranial Wound |
| 852.2* | Subdural Haemorrhage Following Injury Without Mention of Open Intracranial Wound |
| 852.4* | Extradural Haemorrhage Following Injury Without Mention of Open Intracranial Wound |
| 853.0* | Other and Unspecified Intracranial Haemorrhage Following Injury Without Mention of Open Intracranial Wound |
| 958.2 | Secondary and Recurrent Haemorrhage |
| 990.4 | Transfusion of Packed Cells |
| 997.02 | Iatrogenic Cerebrovascular Infarction or Haemorrhage |
| 998.11 | Haemorrhage Complicating A Procedure |
